# Acute Cardiovascular Effects of Simultaneous Energy Drink and Alcohol Consumption in Young Adults: A Review of Case Reports

**DOI:** 10.3390/pediatric16030052

**Published:** 2024-07-29

**Authors:** Victor Azarm, Jan-Philipp Link, Guido Mandilaras, Pengzhu Li, Robert Dalla-Pozza, André Jakob, Nikolaus Alexander Haas, Felix Sebastian Oberhoffer, Meike Schrader

**Affiliations:** Department of Pediatric Cardiology and Intensive Care, University Hospital of Munich, Ludwig-Maximilians-University, 81377 Munich, Germany

**Keywords:** energy drink, alcohol, caffeine, case report, cardiovascular, adverse effects, arrhythmia, death, prevention, atrial fibrillation, young adults

## Abstract

(1) Background: The aim of this review was to identify and summarize adverse cardiovascular health events associated with the simultaneous consumption of energy drinks (ED) and alcohol. Potential prevention strategies and the implementation of research toward the underlying mechanisms for these pathologies were highlighted to emphasize the need for further investigation and to encourage more attention to this field. (2) Methods: The PubMed database was searched for case reports linked with adverse cardiovascular events after simultaneous ED and alcohol consumption. Inclusion criteria were: the reported age of the patient is between 16 and 35 years and confirmed co-consumption of EDs and alcohol. All relevant articles that met the inclusion criteria were fully read and all relevant data was extracted. The extracted data was summarized and presented in this review of cases. (3) Results: In total, 10 cases were identified. The analysis showed that mainly young adults (median age = 24.5 years), in particular men (80%) were affected. The three parts of the cardiovascular system affected were heart rhythm (42%), myocardial function (33%), and coronary arteries (25%). In 3 cases the outcome was fatal. Moreover, preexisting health conditions and/or potential trigger factors were present in 60% of selected cases. (4) Conclusions: This review of case reports suggests that the simultaneous consumption of EDs and alcohol can lead to adverse cardiovascular health events and even incidents with fatal outcomes were reported. Potential trigger factors and preexisting health conditions seem to increase the probability of adverse cardiovascular health events. Consumers should be informed about the potential risks and follow responsible consumption behavior to prevent future health events. More systematic studies are needed to determine the acute effects on the cardiovascular system in young adults.

## 1. Introduction

Energy Drinks (ED) have become a popular beverage choice among young adults in recent years [1,2,3]. ED consumption is primarily driven by the pleasant taste followed by a desire for energy [3]. An ED is defined by the Food and Drug Administration (FDA) as a beverage group that contains caffeine with additional ingredients. EDs typically contain caffeine, sugar, and other stimulants such as taurine, guarana, ginseng, and L-carnitine in various amounts [4]. A study commissioned by the European Food Safety Authority (EFSA) from the Consortium Nomisma-Areté collected consumption data on specific ED consumer groups. The study concluded that out of 7.685 young adults aged 18–29, 13.3% (*n* = 1022) were highly chronic consumers (drinking EDs 4–5 times/week or more). EDs are primarily consumed by children and young adults for their stimulating effects. These beverages can provide a temporary boost in energy, alertness, and concentration. Young people make use of EDs to stay awake longer, enhance performance during sports or physical activities, or simply to combat fatigue [3,5]. 

Multiple studies in minors and adults suggest adverse cardiovascular effects associated with acute ED consumption [5,6,7,8,9,10]. A review published in 2023 by Costantino et al. showed a high prevalence of adverse cardiovascular effects after ED consumption in adults [11]. Acute and chronic ED consumption can lead to arterial hypertension, increased arterial stiffness, and heart rhythm disorders such as QTc prolongation or increased onset of supraventricular extrasystoles [6,10,12,13,14,15,16,17,18,19,20,21]. In extreme cases, this can also lead to fatality, mostly reported in children and young adults [5,10,11,22,23]. Most fatal outcomes are multiorgan failure or rhythm disorders, such as ventricular fibrillation, leading to sudden cardiac death. Moreover, potential triggers and pre-existing health conditions may lead to more severe outcomes.

Alcohol is a ubiquitous and popular substance [24]. It is well studied that alcohol has a negative effect on the cardiovascular system. The acute and chronic consumption of alcohol can lead to ischemic heart disease, hypertension, atrial fibrillation, cardiomyopathy, heart failure, and death [25,26,27]. 

The simultaneous consumption of EDs and alcohol is a very popular choice. In a review conducted by Striley et al., the oldest age group (29–35 years) notably associates ED consumption with alcohol [3]. About 70% of young adults who consume EDs reported mixing EDs with alcohol on a regular basis [1]. To the best of our knowledge, the impact of simultaneous ED and alcohol consumption on the cardiovascular system has not been systematically investigated yet. However, adverse cardiovascular health events after simultaneous ED and alcohol consumption have already been indicated in several reviews focusing on the effects of either EDs or alcohol on the cardiovascular system [7,10,11,28,29]. An overview of the most relevant review articles can be found in Table 1. 

The aim of this review was to identify and summarize the adverse cardiovascular health events associated with the simultaneous consumption of EDs and alcohol. Moreover, the potential impact of additional trigger factors as well as preexisting health conditions was analyzed. 

## 2. Materials and Methods

### 2.1. Eligibility Criteria

#### 2.1.1. Inclusion Criteria

Two researchers performed a systematic literature research. For inclusion, a case report must meet the following criteria: (1) the reported age of the patient is between 16 and 35 years and (2) confirmed co-consumption of EDs and alcohol. 

#### 2.1.2. Exclusion Criteria

Exclusion criteria were the following: the inclusion criteria were not met; repeated publication of the same study population.

### 2.2. Information Sources

We conducted an extensive search of the PubMed database up to the 31 December 2023, analyzed the included case reports and translated articles if necessary. We extracted all relevant data and summarized it in this review of cases.

### 2.3. Search Strategy 

The applied PubMed database search strategy is visualized in Table 2. We performed a search for the most related text words (TW) individually or in combination as follows: “Energy Drink*” and “ethanol*” or “alcohol*” or “vodka*” and “cardiovascular*” or “heart*” or “arrhythmia*”. An overview of the search strategy can be found in Table 2.

### 2.4. Selection Process

Two researchers independently assessed the titles and abstracts found through the search. They thoroughly reviewed all records, relevant articles, and reports and ensured that they met all inclusion criteria. Texts that did not meet these criteria were excluded. Disagreements between researchers were resolved through discussion involving a third researcher to reach a final decision.

### 2.5. Record Selection

**Figure 1:**
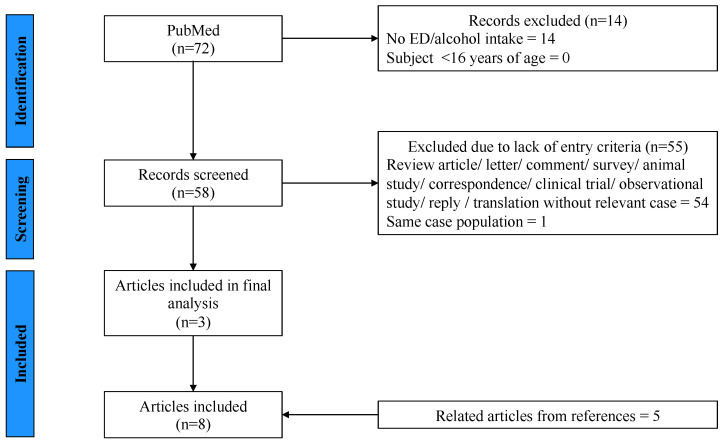
Flow Diagram of Literature Search and Selection Process.

Initially, 72 records were identified through our PubMed database search. 14 records were excluded after reviewing titles and abstracts. An article in Swedish was translated by a native-speaking colleague of our department. The 58 remaining articles were fully reviewed by two independent researchers and selected according to the given inclusion and exclusion criteria. Ultimately, 3 articles with relevant cases were extracted. In addition, 5 cases were identified by screening the literature of the full-text articles from the initial search (Figure 1).

## 3. Results

### Summary of Included Cases

Table 3 provides an overview of the 10 selected cases of adverse cardiovascular events linked to simultaneous ED and alcohol consumption. For each case, we extracted the publishing authors, year of publication, age and sex of the individual, clinical presentation, quantities and type of consumed ED and alcohol, abnormal results, preexisting medical conditions, potential triggers, treatment, and outcome. 

**Figure 2:**
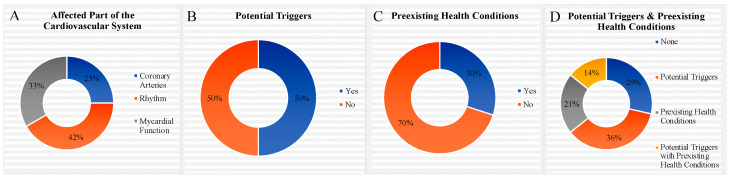
Affected Part of the Cardiovascular System, Potential Triggers and Preexisting Health Conditions Associated with Adverse Cardiovascular Events after the Simultaneous Consumption of EDs and Alcohol (n = 10) (**A**) Affected Part of the Cardiovascular System (Coronary Arteries/Heart Rhythm/Myocardial Function) (**B**) Potential Triggers (Marijuana/Smoking/Chewing Tobacco/Physical Activity/Medication Intake) (**C**) Preexisting Health Conditions (ADHD, Asthma, Allergies/Family History of Coronary Artery Disease, Hypertension/Brugada Syndrome) (**D**) Potential Triggers & Preexisting Health Conditions.

Most of the subjects were young (median age = 24.5 years; range = 16 to 32 years) and male (n = 8). The majority of the patients was discharged in a stable condition; 3 subjects had a fatal outcome. Although a link to EDs and alcohol is assumed, a clear cause of death could not be identified in any of the three cases. The subjects reported the following symptoms: collapse (40%), nausea/emesis/vomiting (40%), chest pain (20%), palpitations (30%), intoxication (10%), head trauma (10%), sleep deprivation (10%). The most popular mixture was different EDs with Vodka (60%). The diagnostic tests revealed: ventricular fibrillation (30%), ST-Segment elevation (30%), pulmonary edema (30%), coronary artery pathology (20%), and atrial fibrillation (30%). Interventions had to be performed in 40% of the subjects. One subject converted spontaneously to sinus rhythm, and the other after administration of intravenous fluid. Preexisting health conditions such as ADHD or arterial hypertension were present in two of the cases. In one case the preexisting health condition Brugada Syndrom was unveiled. Potential additional triggers such as marijuana or tobacco were identified in half of the subjects. Figure 2 summarizes the affected parts of the cardiovascular system, the potential trigger factors and preexisting health conditions that were described in the included case reports.

## 4. Discussion

This review of case reports highlights a correlation between the simultaneous consumption of EDs and alcohol in young adults and adverse cardiovascular health events. Interestingly, most of the patients were male. This is not surprising, as EDs are aggressively marketed towards this peer group by the industry [37]. These marketing efforts often tout benefits such as increased alertness and physical performance, which, when combined with the fatigue-inducing effects of alcohol, create a compelling appeal for simultaneous consumption [38]. 

### 4.1. Effects of EDs on the Cardiovascular System

EDs typically contain a mixture of ingredients, including caffeine, sugar or artificial sweeteners, amino acids such as taurine, guarana, and various B vitamins [39,40,41]. Among these ingredients, caffeine plays a crucial role in the development of adverse cardiovascular events [39,42]. Caffeine especially in high amounts, as found in EDs, is associated with cardiac arrhythmias, prolonged QT interval, ventricular arrhythmias, cardiac arrest, cardiomyopathy, myocardial ischemia, infarction, aortic dissection, and even death [11,40,43]. Of note, these adverse events are often associated with high acute ED intake. Costantino et al. [11] reviewed the reported effects of acute or chronic consumption of energy drinks on human health. Remarkably, the analysis revealed a high prevalence of cardiovascular effects. Of the total study population, 41 cases (47.7%) had adverse cardiac events, including 9 cases of cardiac arrest and 3 deaths. Moderate ED consumption, i.e., 1 can of sugar-sweetened ED (355-mL), is associated with an increased workload on the heart, caused by heightened blood pressure, elevated heart rate, amplified cardiac output, and elevated workload of the heart [42,44]. In addition, the synergistic effect of caffeine with other stimulants such as taurine and guarana, which are found in energy drinks, may enhance these cardiovascular effects. The main component of guarana is caffeine, enhancing the effects mentioned above [45]. The exact impacts of taurine on cardiovascular health is not yet fully understood and reliable data is lacking [37,46,47]. The long-term consequences have also not been sufficiently investigated yet [39].

### 4.2. Effects of Alcohol on the Cardiovascular System

The harmful effects of alcohol on the cardiovascular system are well studied and have been shown in numerous studies and reviews. Alcohol has a diverse and complex relationship with cardiovascular diseases [25]. M. Roereckel subdivided the cardiovascular consequences of alcohol consumption into the following subcategories: atrial fibrillation, ischemic heart disease, hypertension, stroke, and heart failure [25]. Interestingly, the development of atrial fibrillation is particularly high in men and in consumers with high levels of acute alcohol consumption [48]. This drinking pattern can particularly be found in young male adults [1,49,50]. The phenomenon of binge-drinking-alcohol-induced-atrial-fibrillation is also known as “holiday heart syndrome” [51,52]. The relationship between ischemic heart disease and alcohol is described as U-shaped. Both, abstinence and heavy drinking, but not moderate consumption, are associated with a higher risk of ischemic heart disease [53]. Alcohol is associated with decreasing blood pressure 12 h after consumption but increasing it afterwards. Chronic alcohol consumers often tend to have elevated blood pressure [54]. A recently published study has also found that in young male patients, already diagnosed with arterial hypertension, excessive consumption of alcohol can lead to more uncontrolled arterial hypertension [55]. 

Putalaa has found that young adults in particular are at increased risk of developing stroke. The reasons for this are manifold and also due to changes in consumer behavior (e.g., excessive alcohol consumption) in young adults [56]. All of the above-mentioned cardiovascular diseases increase the risk of heart failure. Several meta-analyses and reviews have shown that alcohol is directly and indirectly related to heart failure [14,57,58,59].

### 4.3. Effects of EDs and Alcohol on the Cardiovascular System

The cases presented underscore the cardiovascular risks associated with the simultaneous consumption of EDs and alcohol. Individuals consuming this combination experienced severe arrhythmias such as ventricular and supraventricular fibrillation, coronary artery diseases such as occlusion of an artery by a thrombus or coronary vasospasms, heart failure, and even death (Table 3). Unfortunately, the literature is relatively vague about the severity of the levels of alcohol and EDs caused by the drinks.

A literature review published in 2013 on ED consumption behavior and its consequences found that risky driving, riding with an intoxicated driver, and being taken sexually advantage of significantly rise when EDs are combined with alcohol [3]. In addition, the overall health risks seem to increase when a stimulant (ED) and a depressant (alcohol) are combined. Young adults experience fewer intoxication symptoms such as headache, dry mouth, weakness, and incoordination which pose a huge risk for alcohol-related injury and excessive intoxication [2]. Due to the small but growing body of evidence on the cardiovascular effects of the combination of EDs and alcohol, the exact pathogenesis of these symptoms, adverse cardiovascular effects, and outcomes is still unclear. Pennay et al. reviewed the literature and summarized the three main effects of this combination: (A) The mixture leads to increased diuresis and thus to dehydration [60]. This can lead to electrolyte (e.g., sodium, potassium, calcium) imbalances resulting in nausea, vomiting, and ultimately to arrhythmia, vasospasms, and heart failure. Potentially enhanced when preexisting health conditions and/or potential triggering mechanisms are present. (B) Conflicting signals to the nervous system result in cardiovascular issues (e.g., palpitations, sleep disorders). (C) Masking of the intoxicating effects of alcohol leads to impaired judgment (e.g., risky behavior and poor decision making). All these effects especially from (B) and (C) could be observed in the history of all cases. Rhythm disturbances, such as ventricular- and supraventricular fibrillation were reported in 6 out of 10 cases. This is partly due to the individual effects of EDs and alcohol, and partly due to a combination of the following as described above. Reason (A) in particular has a key effect, while reasons (B) and (C) reinforce this effect. In one of 3 fatal cases, ventricular fibrillation occurred beforehand. This shows that in extreme cases the beverage mix can also lead to fatality. Unfortunately, in the other cases, there is no clear statement on the cause of death. 

### 4.4. Prevention of Adverse Health Events Associated with Simultaneous ED and Alcohol Consumption

Young adults represent the main target group of the ED and alcohol user market. The individual effects of EDs and alcohol are well studied, yet young adults drink alcohol and EDs regularly and in problematic quantities [61]. It’s important to consider not only the immediate cardiovascular effects but also the broader social and cultural factors influencing the consumption of EDs and alcohol together. Social norms, peer pressure, and the acceptance of binge drinking culture play significant roles in shaping young adults’ behaviors regarding substance use. Often, the consumption of EDs and alcohol is intertwined with social activities, parties, and nightlife, where individuals may feel compelled to engage in excessive drinking to fit in or enhance their social experience [29]. Furthermore, the accessibility and affordability of EDs and alcohol contribute to their widespread use, particularly among young adults. The convenience of purchasing these beverages from supermarkets, convenience stores, and vending machines facilitates their consumption, sometimes without adequate consideration of the associated health risks. 

Physicians should warn and discourage their patients from drinking EDs and alcohol simultaneously, especially those with preexisting health conditions and potential trigger factors. Education and awareness campaigns targeting both individuals and communities are essential for addressing these issues. By promoting healthier lifestyle choices and debunking myths surrounding the perceived benefits of combining EDs and alcohol, such campaigns can empower young adults to make informed decisions about their health and well-being. Ultimately, collaboration between healthcare professionals, policymakers, educators, and industry stakeholders is crucial for implementing evidence-based interventions, such as stricter regulations on advertising and marketing practices, restrictions on sales to minors, following the example of many European countries (e.g., Poland, Latvia and Lithuania), and promoting responsible drinking behaviors [62]. Recently, foodwatch, the Society of European Pediatric Cardiologists, as well as experts from the World Health Organization and the Federation of German Consumer Organizations once again advocated for an age limit on the sale of EDs in Germany [63]. To shed more light on this matter our department will conduct the first study systematically investigating the effects of simultaneous ED and alcohol intake on the cardiovascular system of young adults. The “Rhythm of the Night-Study” started collecting data in January 2024. (DRKS00031179)

### 4.5. Limitations

The main limitations are the relatively small number of cases (n = 10) and the lack of clinical information. Medical professionals should be sensitized to adverse cardiovascular events after simultaneous ED and alcohol consumption and should publish corresponding. Some relevant information such as the consumed amounts of EDs and/or alcohol, information about preexisting health conditions, and the exact cause of death were often not included in the case description. Also, some of the information was potentially withheld by the patients (e.g., co-consumption of illegal substances). Therefore, no difference was made between the types and brands of energy drinks (e.g., Monster, Redbull) or alcoholic beverages (e.g., vodka, beer), which may contain varying percentages of stimulants and alcohol. Due to the lack of data available, it was also not possible to come to a clear conclusion if the additional trigger factors have exacerbated, precipitated, or even provoked the symptoms noted.

## 5. Conclusions

In conclusion, this review underscores the concerning correlation between the simultaneous consumption of EDs and alcohol among young adults and its adverse cardiovascular health implications. The analysis of case reports reveals a pattern of severe cardiovascular events, including arrhythmias, coronary artery diseases, and even fatalities, predominantly affecting young males. While the exact mechanisms remain unexplored, a combination of factors such as dehydration, electrolyte imbalances, conflicting nervous system signals, and impaired judgment likely contribute to these outcomes. Social and cultural influences, alongside the accessibility and affordability of these beverages, further exacerbate the risks associated with their concurrent use. Addressing this issue requires multifaceted interventions, including education campaigns, stricter regulations, and collaborative efforts among healthcare professionals, policymakers, and industry stakeholders. Additionally, systematic studies are needed to determine the acute effects of simultaneous ED and alcohol consumption on the cardiovascular system in young adults, providing crucial insights for targeted prevention strategies and enhancing public health outcomes.

## Figures and Tables

**Table 1:** Overview of the most relevant review articles.

Author, Year	Title	Journal	Population	Key Findings
Levy et al., 2019 [7]	European Cardiac Arrhythmia Society Statement on the cardiovascular events associated with the use or abuse of energy drinks	Journal of Interventional Cardiac Electrophysiology	Adults, adolescents and children	Mixing EDs with alcohol is common among young drinkers and can lead to increased alcohol intake, reduced perception of intoxication, and risky behaviors. Regular ED consumers are also more likely to use illicit drugs
Li et al., 2023 [10]	Energy Drinks and Adverse Health Events in Children and Adolescents: A Literature Review	Nutriens	Children, adolescents	Adverse cardiovascular events occur more likely if additional trigger factors are present such as co-ingestion of alcohol
Constantino et al., 2023 [11]	The Dark Side of Energy Drinks: A Comprehensive Review ofTheir Impact on the Human Body	Nutriens	Adults, adolescents	Adverse cardiovascular effects have been found with the simultaneous use of other substances such as alcohol
Goldfarb et al., 2014 [28]	Review of Published Cases of Adverse Cardiovascular Events After Ingestion of Energy Drinks	The American Journal of Cardiology	Adults, adolescents and children	ED consumption linked to cardiovascular events, especially with heavy intake or combined with alcohol/drugs (alcohol). Increased risk of arrhythmias, cardiac arrest, and severe chest pain.
Ehlers et al., (2019) [29]	Risk assessment of energy drinks with focus on cardiovascular parameters and energy drink consumption in Europe	Food and Chemical Toxicology	Adults	EDs and alcohol combined increase the risk of arrhythmias and masking intoxication effects, leading to risky behaviors. Case reports suggest cardiovascular and renal adverse effects. More research needed to determine safe consumption levels

**Table 2:** Search Strategie.

Search Number	Title 2
Queries in PubMed	
#1	Search (Energy Drink*[TW] AND (ethanol* or alcohol* or vodka*))
#2	Search (cardiovascular* or heart* or arrhythmia*)
#3	#1 AND #2

* Wildcard for capture variations of search terms. TW: text word.

**Table 3:** Summary of Included Cases Reporting Adverse Cardiovascular Events after the Simultaneous Consumption of Energy Drinks and Alcohol.

Author, Year	Age, Sex	Clinical Presentation	ED Consumption Behavior	Alcohol Consumption Behavior	Abnormal Findings	Potential Triggers	Preexisting Health Conditions	Treatment	Outcome
Rutledge, M. (2012) [30]	24, M	Collapse	Red Bull (80 mg caffeine, 1000 mg taurine)	Vodka (amount not specified)	Ventricular fibrillation, ST-segment elevation, bilateral pulmonary edema	None	Brugada Syndrome	Intubation, amiodarone, naloxone, epinephrine, defibrillation, AICD placement	Stable discharge after two days
Benjo, A. M. (2012) [31]	24, M	Nausea, emesis, palpitations, chest pain	Unknown quantities and type of EDs	Vodka (amount not specified)	ST-segment elevation, thrombus in left main coronary artery, congestive heart failure after coronary angiography	Marijuana	None	Emergent coronary bypass graft surgery, warfarin	Stable discharge on warfarin
Di Rocco, J. R. (2011) [32]	16, M	Intoxication, vomiting, head trauma	Unknown quantities of Red Bull	Vodka (amount not specified)	Atrial fibrillation	Amphetamine, dextroamphetamine (Adderall XL), 30 mg daily; montelucast (Singulair), 10 mg daily; loratadine (Claritin), 10 mg daily; and doxycycline, 100 mg daily	ADHD, asthma, allergies	IV fluids, cardiac monitoring, spontaneous conversion to sinus rhythm	Normal sinus rhythm, no recurrence of arrhythmia, stable discharge
Sattari, M. (2016) [33]	28, M	Vomiting of blood, palpitations	Daily two “Monster energy” (320 mg caffeine)	2–3 Beer	Atrial fibrillation with rapid ventricular response	Chewing tobacco	None	PPI, beta-blocker	Stable discharge
Zacher, J. (2018) [34]	25, M	Severe chest pain	>8 cans of different EDs (>2 L)	Strong liquor (amount not specified)	ST-segment elevation, proximal LAD dissection	Smoking	Arterial hypertension, family history of coronary artery disease	Coronary angiography, drug eluting stents	Left ventricular dysfunction, stable discharge after treatment
Mattioli, A. V. (2018) [35]	26, M	Anxiety, nausea, palpitations	600 mL ED	Alcoholic beverage (30 g alcohol)	Slightly dilated left atrial volume, high-rate AF at 170 bpm	None	None	Spontaneous conversion to sinus rhythm	Stable discharge with negative 12-month follow-up
Osman, H. (2019) [36]	32, M	Lack of sleep, palpitations	48 cans (1 can = 250 mL) of ED XXL Vodka Mix, 32 mg/100 mL caffeine	48 cans of XXL Vodka Mix containing vodka 10.2% vol	Ventricular fibrillation, elevated CK, CK-MB, troponin	None	None	In-hospital cardiac arrest, defibrillation, coronary care unit admission	Stable discharge after three days
Lehtihet, M. (2006) [23]	19, F	Death	Six drinks of Red Bull	Vodka (blood alcohol concentration postmortem = 0.87‰)	Forensic examination revealed a picture of severe pulmonary edema with hemorrhagic features	None	Unknown	None	Fatal, found dead in her bed the morning after
Lehtihet, M. (2006) [23]	31, F	Weak pulsations in the carotid artery, required assisted ventilation, pulselessness, in hospital: ventricular fibrillation	Unknown quantities of Red Bull	Vodka (blood alcohol concentration postmortem = 0.63‰)	Ventricular fibrillation, forensic examination revealed only a slight deposition of connective tissue in the heart muscles and a slight fatty degeneration of the liver	Physical activity (dancing), screening for intake of medicines and narcotics was negative	Unknown	Resuscitation on scene, advanced CPR during transport to hospital, in hospital: defibrillation a total of 15 times	Fatal
Lehtihet, M. (2006) [23]	18, M	Collapsed, lifeless	a couple of cans of Red Bull a day for a week	Unknown type of alcohol (blood alcohol concentration postmortem = 0,59‰, urine alcohol concentration postmortem = 0.80‰)	Forensic medical examination revealed pronounced cerebral edema, pronounced pulmonary edema and mild to moderate diffuse connective tissue deposition in the heart muscles	None	Unknown	CPR	Fatal

ADHD: Attention deficit hyperactivity disorder, AICD: Automatic implantable cardioverter defibrillator, IV: Intravenous, PPI: Proton-Pump-Inhibitors, AF: Atrial fibrillation, LAD: Left anterior descending, CK: Creatin Kinase, CK-MB: Creatin Kinase-Muscle Brain, M: Male, ED: Energy drink, F: Female, CPR: Cardiopulmonary resuscitation.

## Data Availability

No datasets were generated or analyzed during the current study.
